# High-Output Bile Leak Following AAST Grade III Liver Injury Resolved With Endoscopic Stenting: A Case Report

**DOI:** 10.7759/cureus.94528

**Published:** 2025-10-14

**Authors:** Yoichi Miyaoka, Shingo Shimada, Shinya Ueki, Tomoyuki Takagi, Akinobu Taketomi

**Affiliations:** 1 Department of Surgery, Wakkanai City Hospital, Wakkanai, JPN; 2 Department of Surgery, Otaru General Hospital, Otaru, JPN; 3 Department of Surgery, Asahikawa Kosei General Hospital, Asahikawa, JPN; 4 Department of Gastroenterological Surgery 1, Hokkaido University Graduate School of Medicine, Sapporo, JPN

**Keywords:** bile leak management, biloma, blunt liver injury, erbd (endoscopic retrograde biliary drainage), ercp (endoscopic retrograde cholangiopancreatography), nonoperative management

## Abstract

Nonoperative management (NOM) has become the standard of care for hemodynamically stable blunt liver trauma; however, biliary complications may delay recovery. We report the case of a previously healthy 20-year-old man who sustained an AAST Grade III liver laceration in a motor vehicle collision and subsequently developed a high-output intrahepatic bile leak. An ultrasound-guided percutaneous drain initially produced 880 mL/day, with the output remaining elevated at approximately 450 mL/day. Endoscopic retrograde cholangiopancreatography (ERCP) demonstrated extravasation from a posterior branch of the right intrahepatic duct. Placement of a 7-Fr transpapillary plastic stent (endoscopic retrograde biliary drainage, ERBD) resulted in rapid reduction and eventual cessation of bile output, with closure confirmed on tubography and drain removal on hospital day (HD) 42.

The patient recovered without surgical intervention and remained well on follow-up. This case illustrates that clinically significant bile leakage can occur even after moderate liver injury and highlights a step-up management strategy, percutaneous drainage followed by ERCP, that can be effectively applied in resource-limited community hospitals.

## Introduction

Blunt liver trauma is among the most common abdominal injuries, and current practice favors nonoperative management (NOM) for hemodynamically stable patients [1]. This paradigm shift from routine laparotomy to selective NOM has been supported by advances in imaging, intensive monitoring, and interventional radiology, resulting in high success rates among stable cases. The rationale for NOM is to avoid unnecessary surgery and its associated morbidity while ensuring timely intervention if bleeding, peritonitis, or other complications develop.

Within structured NOM pathways, delayed hepatobiliary complications, particularly biloma and bile leak, occur in approximately 3%-7% of cases and may prolong hospitalization or require additional procedures [2]. The American Association for the Surgery of Trauma (AAST) Organ Injury Scale (OIS) standardizes liver injury grading and guides both prognosis and management decisions [3,4]. Although high-grade injuries (AAST IV-VI) involving major vascular or biliary disruption are known to carry higher risks of bile leakage, clinically relevant leaks may also occur after moderate injuries (AAST III) when lacerations extend into segmental bile ducts [2].

Management of post-traumatic bile leakage depends primarily on output volume, infection risk, and patient stability. Most minor leaks resolve spontaneously with conservative care, whereas persistent or high-output leaks require decompression of the biliary system. Traditionally, surgery was performed in such cases; however, endoscopic retrograde cholangiopancreatography (ERCP) has become a minimally invasive alternative that achieves high closure rates and aligns with NOM principles [5-7].

This report describes a case of AAST Grade III liver laceration complicated by a high-output intrahepatic bile leak, successfully managed with a step-up approach (percutaneous drainage followed by transpapillary stenting). The case highlights that significant bile leakage can occur even in moderate-grade injuries and demonstrates the feasibility of modern minimally invasive management strategies in resource-limited community hospitals.

## Case presentation

A previously healthy 20-year-old man was transferred to our emergency department approximately 1.5 hours after a motor vehicle collision. On arrival, he was hemodynamically stable (blood pressure 118/72 mmHg, pulse 86 bpm) and complained of right upper quadrant pain. Admission laboratory evaluation showed leukocytosis and elevated transaminases without clinically significant anemia or coagulopathy. The remaining laboratory findings were within or near the lower limits of our institutional reference ranges and were consistent with a physiological response to blunt trauma (Table 1).

**Table 1:** Admission laboratory values Reference ranges reflect adult male values from our institutional laboratory. Note: Units are shown in SI or commonly accepted conventional units. Reference ranges may vary by laboratory.

Laboratory test	Result (units)	Reference range
White blood cells (WBCs)	22.8 × 10^9^/L	4.0-10.0 × 10^9^/L
Red blood cells (RBCs)	4.31 × 10^12^/L	4.5-5.9 × 10^12^/L (male)
Hemoglobin (Hb)	13.0 g/dL	13.5-17.5 g/dL (male)
Hematocrit (Hct)	38.5%	41%-53% (male)
Platelets (PLTs)	280 × 10^9^/L	150-400 × 10^9^/L
Mean corpuscular volume (MCV)	89.3 fL	80-100 fL
Mean corpuscular hemoglobin (MCH)	30.2 pg	27-33 pg
Mean corpuscular hemoglobin concentration (MCHC)	33.8 g/dL	32-36 g/dL
Red cell distribution width (RDW)	12.7%	11.5-14.5%
Mean platelet volume (MPV)	9.0 fL	7.5-11.5 fL
Total protein (TP)	6.2 g/dL	6.6-8.3 g/dL
Albumin (ALB)	4.3 g/dL	3.5-5.0 g/dL
Total bilirubin (TBil)	0.7 mg/dL	0.2-1.2 mg/dL
Aspartate aminotransferase (AST)	274 U/L	0-40 U/L
Alanine aminotransferase (ALT)	215 U/L	0-41 U/L
Gamma‑glutamyl transferase (GGT)	19 U/L	8-61 U/L
Lactate dehydrogenase (LDH)	644 U/L	120-246 U/L
Creatine kinase (CK)	337 U/L	30-200 U/L
Prothrombin time (PT)‑international normalized ratio (INR)	1.08	0.9-1.1
Prothrombin time (PT)	12.8 s	11-14 s
Activated partial thromboplastin time (APTT)	24.0 s	25-35 s
Fibrinogen (Fib)	199 mg/dL	200-400 mg/dL
Antithrombin activity (AT‑III)	123%	80%-120%
Fibrin/fibrinogen degradation products (FDPs)	12.1 µg/mL	<5.0 µg/mL

**Figure 1:**
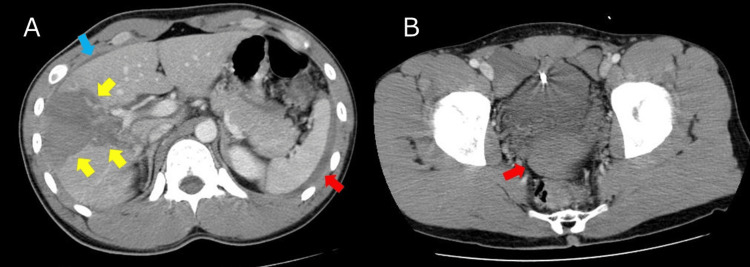
Contrast-enhanced CT on admission (A) Axial contrast-enhanced CT demonstrated a deep parenchymal laceration in the right anterior hepatic sector (yellow arrows) with a subcapsular hematoma along the hepatic surface (blue arrow). There is also a perisplenic intraperitoneal hemorrhage (red arrow). (B) Axial CT of the pelvis showed hemoperitoneum accumulating in the pelvic cavity (red arrow). No active contrast extravasation is observed, and the injury was classified as AAST Grade III.

Contrast-enhanced computed tomography (CT) on admission demonstrated a deep laceration in the right anterior hepatic sector without evidence of major vascular injury or active contrast extravasation (Figure 1A). A small pelvic hemoperitoneum was also noted (Figure 1B).

The injury was classified as AAST-OIS Grade III.

Because air transfer was precluded by weather conditions and ground transfer to a tertiary center would have required more than two hours, the patient was managed nonoperatively at our community hospital, which is equipped for surgery but lacks on-site interventional radiology and has limited blood stock. He was admitted to the intensive care unit for close observation during the initial phase of NOM. Empiric intravenous antibiotics were initiated and continued for seven days. His vital signs and hemoglobin levels remained stable throughout early observation.

**Figure 2:**
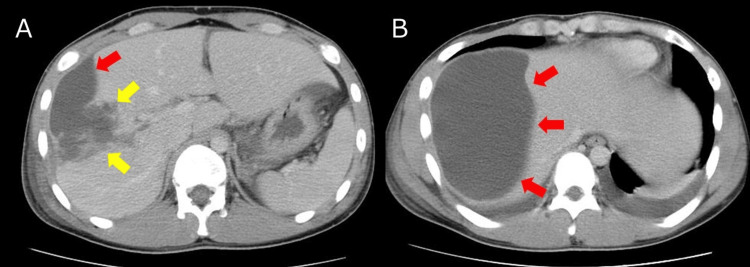
CT on hospital day 14 (biloma) (A) Axial CT showed an encapsulated perihepatic fluid collection adjacent to the laceration (yellow arrows). (B) Large perihepatic fluid consistent with biloma (red arrows).

On hospital day (HD) 14, the patient developed a fever of 39 °C. Follow-up CT demonstrated an encapsulated perihepatic fluid collection adjacent to the laceration, consistent with a biloma (Figures 2A-2B).

**Figure 3:**
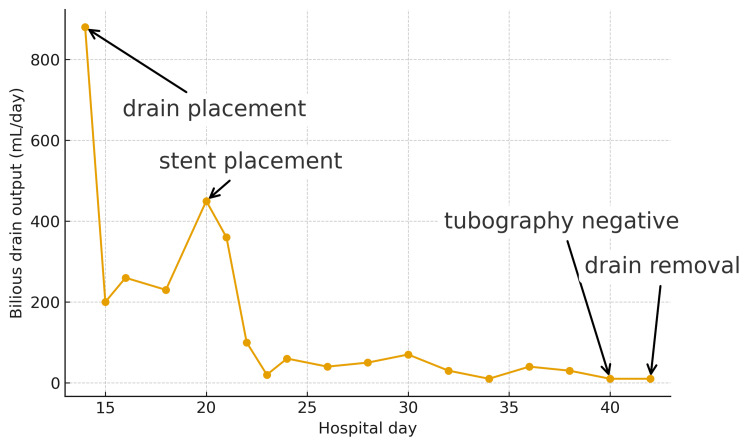
Daily bile output and timeline of interventions Time series of bilious drain output (mL/day) plotted against hospital day. Key events are annotated with horizontal labels and arrows to the corresponding data points: drain placement, stent placement, tubography negative, and drain removal. Output peaked at 880 mL/day immediately after diagnosis and drainage, remained high around 450 mL/day before stent placement, and then declined steadily to near-zero by the time tubography confirmed no extravasation, after which the drain was removed.

Ultrasound-guided percutaneous drainage was performed, yielding bilious fluid, and the catheter was left in place for continuous drainage. The initial daily output was 880 mL. To illustrate the subsequent clinical course, the day-by-day trend of bile output and the timing of key interventions are shown in Figure 3.

**Figure 4:**
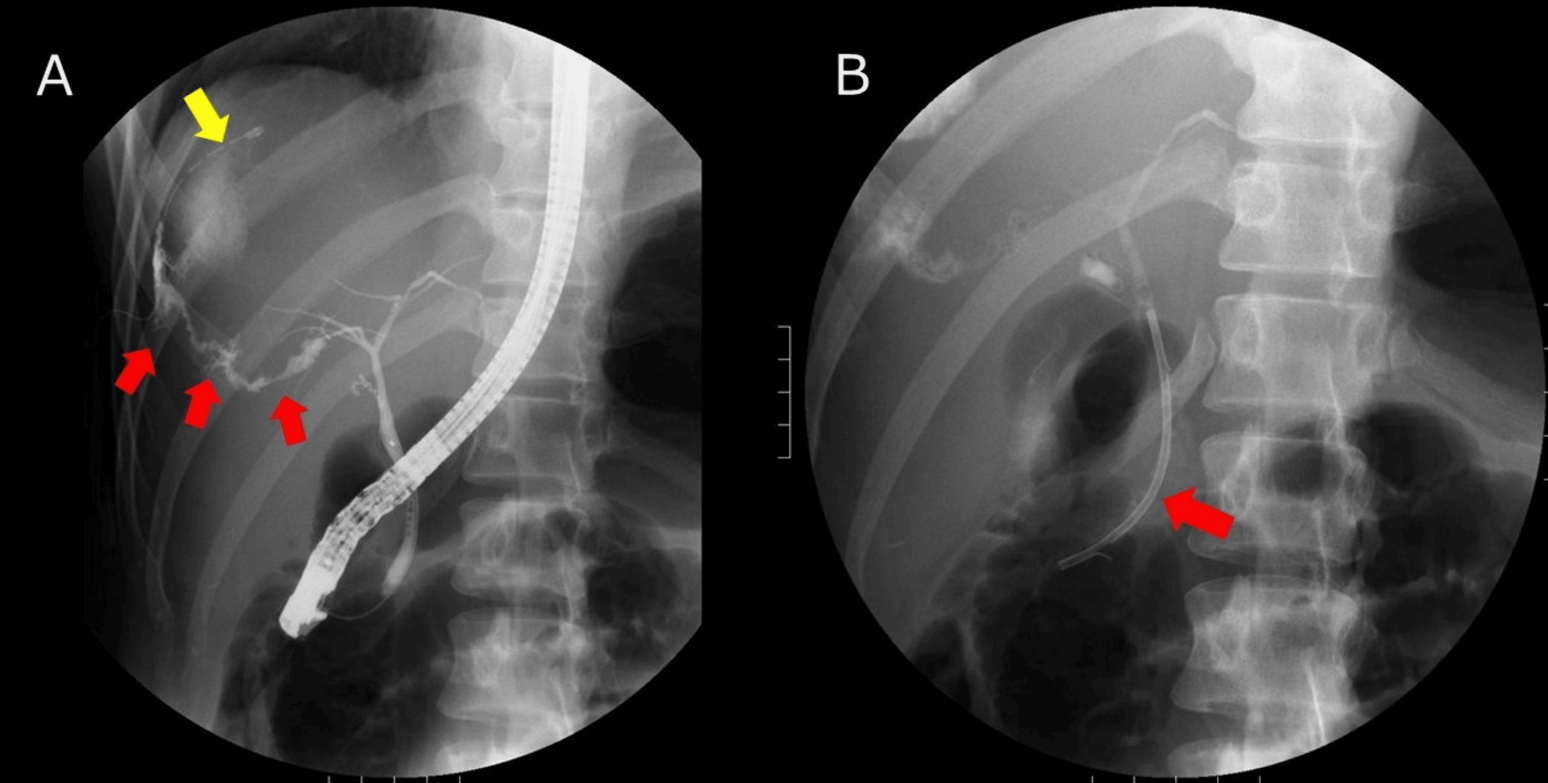
Endoscopic retrograde cholangiopancreatography (A) Cholangiography demonstrated contrast extravasation from the right posterior sectoral duct arising near the crotch of the right anterior and left hepatic ducts (posterior take-off pattern) (red arrows). Extraluminal contrast tracks away from the biliary tree and pools in the peritoneal cavity (yellow arrows), consistent with bile leaking into the abdomen. (B) A 7-Fr plastic transpapillary biliary stent (endoscopic retrograde biliary drainage, ERBD) is placed across the papilla (red arrow).

Despite adequate drainage, output increased again to approximately 450 mL/day, prompting endoscopic evaluation. ERCP showed extravasation from the right posterior sectoral duct (posterior take-off near the hilar confluence) (Figure 4A). A 7-Fr transpapillary plastic stent was deployed across the papilla to decompress the biliary tree (Figure 4B), and output subsequently declined.

**Figure 5:**
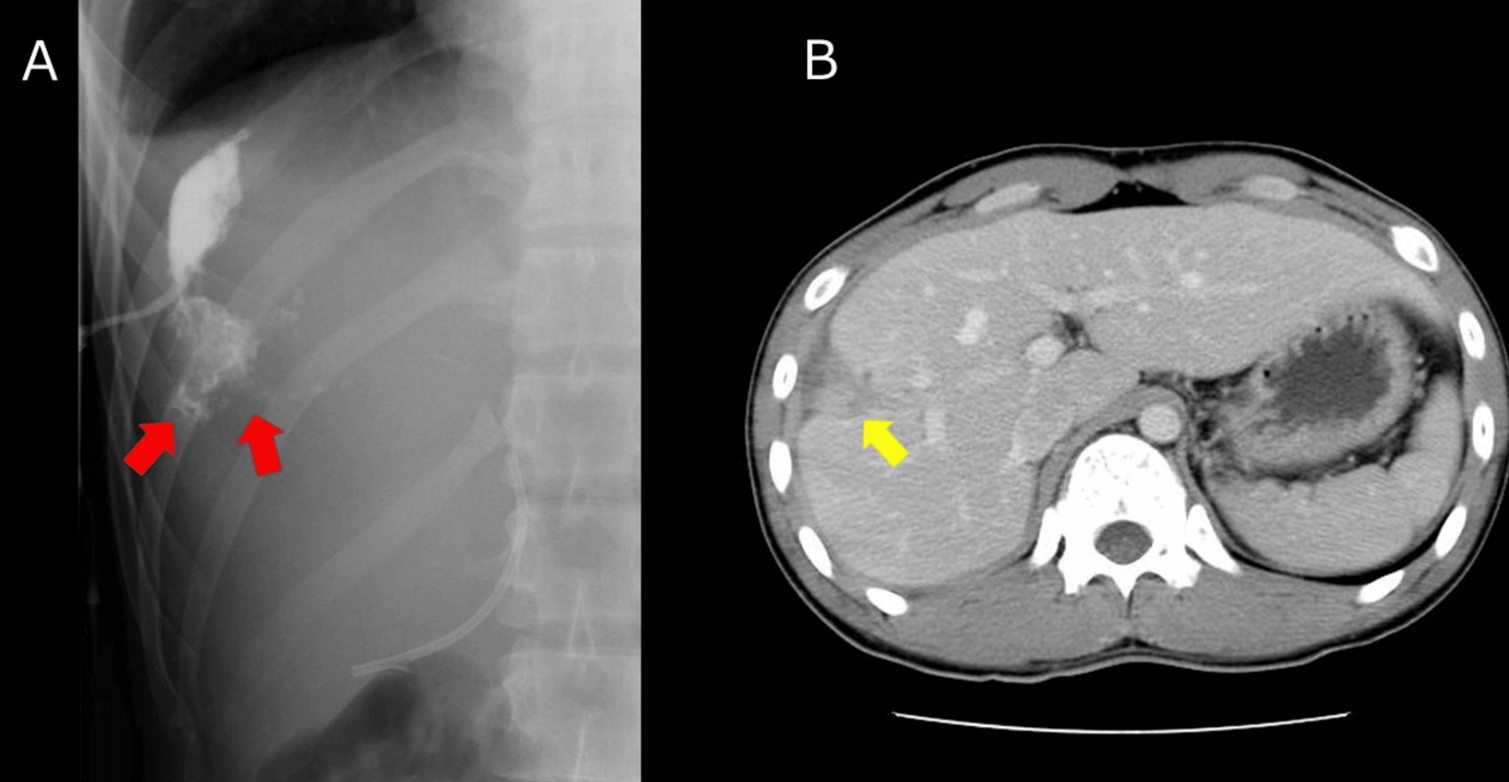
Leak closure and follow-up (A) Tubography via the percutaneous biloma drain on hospital day 40 shows focal pooling of contrast within the fistulous tract only (red arrows) without opacification of the intrahepatic bile ducts, indicating no residual communication. (B) Follow-up contrast-enhanced CT demonstrates complete resolution of the biloma; the site adjacent to the prior laceration is indicated (yellow arrow).

Thereafter, as illustrated in Figure 5, the drain output declined steadily and had ceased by HD 40. Tubography via the percutaneous catheter on that day confirmed the absence of further leakage (Figure 5A). The catheter was removed on HD 42, and the patient was discharged home. Follow-up CT performed 70 days after injury demonstrated complete resolution of the biloma without bile duct dilatation (Figure 5B). The transpapillary stent was subsequently removed electively. The patient remained asymptomatic thereafter.

## Discussion

Clinically significant biliary complications after blunt liver trauma are uncommon but consequential, occurring in approximately 3%-7% of patients managed nonoperatively and often prolonging recovery when present [1,2,8]. Although high-grade injuries (AAST IV-VI) are classically associated with bile duct involvement, this case highlights that moderate injuries (AAST III) can also result in high-output bile leakage when a parenchymal cleft intersects a segmental duct [2-4,8]. Notably, our patient had not undergone embolization, underscoring that non-ischemic mechanisms alone may be sufficient to produce clinically relevant leakage.

Diagnosis in practice is multifactorial. First, contrast-enhanced CT defines the anatomic extent of injury, excludes indications for urgent surgery, and identifies perihepatic collections that may represent biloma. Second, biochemical confirmation adds specificity: a drain-to-serum bilirubin ratio ≥ 3 fulfills the International Study Group of Liver Surgery (ISGLS) definition of bile leak and is widely used in hepatobiliary practice [9]. Third, ductal imaging is pursued when results are likely to alter management. Hepatocyte-specific magnetic resonance cholangiopancreatography (MRCP) can enhance detection and characterization of active traumatic leaks and help distinguish contained from expanding collections [10]. In our case, a localized collection was identified on CT, but the definitive diagnosis of biloma was confirmed by the bilious character of aspirated fluid during percutaneous drainage, after which management was escalated selectively.

Management is best conceptualized as a staged decompression strategy consistent with modern NOM principles. Image-guided drainage addresses symptomatic or enlarging collections and mitigates the risk of sepsis, while persistent or recurrent high-output drainage warrants biliary decompression. Although formal cutoffs are not universally defined, pragmatic thresholds include failure of drainage output to decrease below approximately 200 mL/day after several days of adequate catheter function (extrapolated from post-cholecystectomy algorithms) [11]. Trauma cohorts have similarly identified patients with output ≥300-400 mL/day at diagnosis as most likely to require endoscopic or operative intervention [8]. Our patient exhibited an initial peak of 880 mL/day, a brief decline to around 200 mL/day, followed by a renewed rise into the high-output range, prompting ERCP.

Endoscopic therapy achieves high closure rates with low morbidity in post-traumatic bile leaks and aligns with NOM principles by avoiding laparotomy in stable patients [5-7,12,13]. Plastic transpapillary stents (7-10 Fr) effectively divert bile, with trauma-focused series reporting closure rates approaching 90% [5-7]. Routine sphincterotomy is not mandatory during stent placement and may be reserved for difficult cannulation or the use of large-caliber devices. The choice between endoscopic retrograde biliary drainage (ERBD) and endoscopic nasobiliary drainage (ENBD) should be individualized [5-7,12,13]. ENBD allows direct monitoring of output and facilitates repeat cholangiography but externalizes bile, risking fluid and electrolyte loss as well as patient discomfort. ERBD preserves physiologic bile flow and comfort, offering comparable sealing efficacy when the leak can be spanned, that is, when the distal end of the stent crosses the segmental duct containing the injury, allowing direct internal drainage from the injured segment into the common bile duct. In trauma-related leaks, selective bridging may not always be feasible, and decompression alone often suffices, as in our case, where a 7-Fr ERBD led to progressive cessation of output. Tubography on HD 40 showed no residual leakage, permitting drain removal on HD 42, and the transpapillary stent was electively removed 70 days after injury [12,13]. Typical stent dwell time is 4-7 weeks (extended to 6-12 weeks in complex leaks), with removal following clinical quiescence and imaging or tubographic confirmation of closure [12,13].

We also highlight the role of tubography. Although MRCP was available, bedside tubography via the existing drain was used as a simple, low-risk method to confirm closure without requiring patient transfer or additional contrast CT exposure. The absence of intrahepatic opacification on hospital day 40 supported safe drain removal. In tertiary centers, MRCP may be preferred for comprehensive ductal mapping; however, tubography remains a practical and sufficient approach for confirming closure in appropriately selected, clinically improving patients.

To contextualize our case across different care settings, Table 2 summarizes AAST liver injury grades (I-VI) alongside typical management pathways referenced in the World Society of Emergency Surgery (WSES) 2020 and AAST guidelines. Individual management should always be tailored to patient stability, available resources, and the evolving clinical course.

**Table 2:** AAST liver injury grades and typical management (per WSES 2020 and AAST guidance) This table states the AAST Grades (I-VI) and the corresponding typical management pathways recommended by the World Society of Emergency Surgery (WSES) 2020 guidelines and the American Association for the Surgery of Trauma (AAST). Final management should be individualized to hemodynamic stability, associated injuries, and available resources. NOM: nonoperative management, ERCP: endoscopic retrograde cholangiopancreatography.

AAST grade	Typical injury features	Typical management (WSES/AAST)
I-II	Minor capsular tears or superficial lacerations	NOM in stable patients; observation, analgesia; no routine IR/transfusion
III	Deeper parenchymal laceration without major vascular/biliary disruption	NOM with close monitoring; treat symptomatic collections; escalate for complications
IV	Parenchymal disruption with segmental vessel/duct involvement	NOM in selected stable patients; IR for bleeding as needed; bile leak: drainage → ERCP
V	Major devascularization or juxtahepatic venous injury	Individualize; damage-control surgery if unstable; IR as indicated; bile leak: drainage/ERCP
VI	Hepatic avulsion	Operative management (often non-survivable)

Finally, this case offers a pragmatic systems perspective for community hospitals that may lack immediate operative capacity, a robust blood bank, or on-site interventional radiology. When ERCP is available, a straightforward step-up pathway (initial stabilization and percutaneous drainage followed by ERCP with transpapillary stenting if output remains high) can provide definitive, low-morbidity management without laparotomy. Where ERCP is not available, the same strategy remains feasible through local stabilization and image-guided drainage, followed by planned referral for therapeutic ERCP to achieve closure, as recommended by the WSES 2020 liver trauma guidelines and AAST guidance [1,3,12,13]. Beyond hemodynamic stability, prerequisites for safe local management generally include (i) no active hemorrhage or hollow viscus injury on contrast CT, (ii) absence of diffuse peritonitis or uncontrolled sepsis, (iii) controllable drain output with stable electrolytes and renal function, and (iv) timely access to ERCP either on site or through coordinated transfer. These criteria align with published trauma pathways and outcomes data for delayed biliary complications [1,3,8,12,13].

In summary, this case illustrates that even moderate (AAST III) hepatic injury can result in high-output bile leakage. A structured, minimally invasive step-up approach, anchored by ERCP, can achieve definitive resolution safely, even in resource-limited community settings.

## Conclusions

Even outside tertiary centers, a step-up strategy centered on ERCP can be definitive and potentially life-saving: stabilize the patient, drain percutaneously, and then perform ERCP with transpapillary plastic stenting if high-output leakage persists. In small community hospitals without immediate operative capacity or massive transfusion resources, on-site ERCP (or rapid access via planned referral) can prevent sepsis, avoid surgery, and expedite recovery.
